# Radiomic Features of the Hippocampus for Diagnosing Early-Onset and Late-Onset Alzheimer’s Disease

**DOI:** 10.3389/fnagi.2021.789099

**Published:** 2022-01-26

**Authors:** Yang Du, Shaowei Zhang, Yuan Fang, Qi Qiu, Lu Zhao, Wenjing Wei, Yingying Tang, Xia Li

**Affiliations:** ^1^Department of Geriatric Psychiatry, Shanghai Mental Health Center, Shanghai Jiao Tong University School of Medicine, Shanghai, China; ^2^Alzheimer’s Disease and Related Disorders Center, Shanghai Jiao Tong University, Shanghai, China; ^3^Shanghai Key Laboratory of Psychotic Disorders, Shanghai Mental Health Center, Shanghai Jiao Tong University School of Medicine, Shanghai, China

**Keywords:** early-onset Alzheimer’s disease, late-onset Alzheimer’s disease, hippocampus, radiomics, support vector machine

## Abstract

**Background**: Late-onset Alzheimer’s disease (LOAD) and early-onset Alzheimer’s disease (EOAD) are different subtypes of AD. This study aimed to build and validate radiomics models of the hippocampus for EOAD and young controls (YCs), LOAD and old controls (OCs), as well as EOAD and LOAD.

**Methods**: Thirty-six EOAD patients, 36 LOAD patients, 36 YCs, and 36 OCs from the Alzheimer’s Disease Neuroimaging Initiative (ADNI) database were enrolled and allocated to training and test sets of the EOAD-YC groups, LOAD-OC groups, and EOAD-LOAD groups. Independent external validation sets including 15 EOAD patients, 15 LOAD patients, 15 YCs, and 15 OCs from Shanghai Mental Health Center were constructed, respectively. Bilateral hippocampal segmentation and feature extraction were performed for each subject, and the least absolute shrinkage and selection operator (LASSO) method was used to select radiomic features. Support vector machine (SVM) models were constructed based on the identified features to distinguish EOAD from YC subjects, LOAD from OC subjects, and EOAD from LOAD subjects. The areas under the receiver operating characteristic curves (AUCs) were used to evaluate the performance of the models.

**Results**: Three, three, and four features were selected for EOAD and YC subjects, LOAD and OC subjects, and EOAD and LOAD subjects, respectively. The AUC and accuracy of the SVM model were 0.90 and 0.77 in the test set and 0.91 and 0.87 in the validation set for EOAD and YC subjects, respectively; for LOAD and OC subjects, the AUC and accuracy were 0.94 and 0.86 in the test set and 0.92 and 0.78 in the validation set, respectively. For the SVM model of EOAD and LOAD subjects, the AUC was 0.87 and the accuracy was 0.79 in the test set; additionally, the AUC was 0.86 and the accuracy was 0.77 in the validation set.

**Conclusion**: The findings of this study provide insights into the potential of hippocampal radiomic features as biomarkers to diagnose EOAD and LOAD. This study is the first to show that SVM classification analysis based on hippocampal radiomic features is a valuable method for clinical applications in EOAD.

## Introduction

Alzheimer’s disease (AD), characterized by progressive cognitive dysfunction, is a common neurodegenerative disorder that significantly affects the quality of life of patients (DeTure and Dickson, 2019). AD is clinically classified into early-onset AD (EOAD) and late-onset AD (LOAD) based on the age of symptom onset (Tellechea et al., 2018). A recent study has suggested considerable differences between EOAD and LOAD in etiological and clinical heterogeneity (Ayodele et al., 2021). Compared with LOAD patients, EOAD patients exhibit more aggressive disease progression and an atypical presentation of preserved memory function but focal cortical symptoms such as language, visuospatial, and executive dysfunction (Cacace et al., 2016).

Consistent with the differences in clinical characteristics, EOAD and LOAD patients also exhibit distinctions in neuroimaging findings. Previous structural imaging studies have shown that compared with LOAD patients, EOAD patients present with less atrophy in the hippocampus but more severe atrophy in the neocortex, particularly the parietal and precuneus and posterior cingulate cortices (Moller et al., 2013; Cavedo et al., 2014; Joubert et al., 2016). Furthermore, some resting-state functional magnetic resonance imaging (fMRI) studies have indicated that patients with EOAD exhibit functional disruption between the hippocampus and middle frontal cortex, while LOAD patients show more widely disrupted hippocampal functional connectivity (Park et al., 2017; Li et al., 2018). These findings indicate that AD is a heterogeneous disorder with significant differences between EOAD and LOAD. Therefore, the hippocampus is likely to exert a specific effect on the pathologies of the two subtypes of AD and function as a useful biomarker in the differential diagnosis of EOAD and LOAD.

Radiomics, an emerging imaging analysis method, can objectively and quantitatively describe phenotypic information using advanced imaging features (Gillies et al., 2016). Radiomic features refer to histogram-based features, including skewness and kurtosis, and texture-based features, such as the gray-level cooccurrence matrix (GLCM) and the gray-level run-length matrix (GLRLM), which provide microstructural information unique from that indicated by volumetric measures (Mayerhoefer et al., 2020). Currently, radiomics has been widely applied to MRI and positron emission tomography (PET) as imaging biomarkers of AD (Cai et al., 2020). Recent MRI-based radiomics studies have shown that textural features of the hippocampus are valid to distinguish AD patients from healthy controls (Chaddad et al., 2018; Feng et al., 2018, 2019; Luk et al., 2018; Li et al., 2020). Several studies have suggested that hippocampal texture is superior to volume changes as a predictor of AD (Beheshti et al., 2017; Shu et al., 2021). However, most of the above studies have concentrated on the textural features of the hippocampus in patients with LOAD, and several studies included both EOAD and LOAD patients as a whole AD group, missing an opportunity to identify differences between the two subtypes of AD. No evidence exists regarding the extraction and modeling of radiomic features between EOAD and healthy subjects or between EOAD and LOAD patients.

In this study, we are the first to investigate and validate hippocampus-based radiomic features for diagnosing EOAD patients and young healthy subjects. Additionally, we sought to ascertain hippocampal texture as a good biomarker in patients with LOAD and old healthy subjects. Furthermore, this study is the first to explore and validate hippocampal radiomic features and construct classification models for distinguishing between patients with EOAD and LOAD.

## Methods

### Study Participants

The training and test data used in this study were obtained from the Alzheimer’s Disease Neuroimaging Initiative (ADNI) database^1^. The ADNI was launched in 2003 as a public-private partnership led by the National Institute on Aging (NIA), the Food and Drug Administration (FDA), and National Institute of Biomedical Imaging and Bioengineering (NIBIB). The ADNI aims to aid researchers and clinicians in developing new treatments and monitoring their effectiveness as well as to lessen the time and cost of clinical trials. Up-to-date information can be found at www.adni-info.org. The use of the ADNI data was approved by the institutional review board at each site, and all the participants provided their written consent.

A total of 144 ADNI participants were included in this study 36 EOAD, 36 LOAD, 36 young control (YC), and 36 old control (OC) participants from the ADNI1, ADNI2/GO, and ADNI3 databases. Scans were collected at either screening or baseline visits. First, 36 patients diagnosed with AD onset before the age of 65 years (EOAD) who were enrolled in the ADNI database were eligible for this study. Next, we included 36 patients who were 65 years or older at disease onset (LOAD) and who were 1:1 matched to the EOAD patients by the Clinical Dementia Rating (CDR) Scale. Accordingly, we selected two control groups for each patient group. The controls were matched 1:1 to AD patients for age and sex, thus obtaining a YC group for EOAD (*n* = 36; YC) and an OC group for LOAD (*n* = 36; OC). Furthermore, demographic information, medical history, baseline symptoms, and assessment scale scores were included. The MRI and clinical data were downloaded in June 2021.

Independent external validation data were acquired from the Memory Clinic of Shanghai Mental Health Center (SMHC) between July 2017 and May 2021, and normal control subjects were recruited from the community. A total of 60 participants including 15 EOAD, 15 LOAD, 15 YCs, and 15 OCs were enrolled in this study. Similarly, 15 LOAD patients were also 1:1 matched to the EOAD patients using the CDR Scale, and the controls were matched to AD patients for age and sex. EOAD and LOAD patients were diagnosed by two experienced geriatric psychiatrists. The exclusion criteria included the following: (1) other psychiatric disorders comorbidities; (2) a history of major physical illness, cardiovascular disease, or neurological disorder; (3) substance abuse or dependence; (4) pregnancy. Neuropsychological tests and brain imaging scans were performed in all subjects. The retrospective study was approved by the ethics committee of the Shanghai Mental Health Centre of Shanghai Jiao Tong University School of Medicine, and all the participants provided written informed consent after they were given a description of this study.

### Image Acquisition

Regarding ADNI data, T1-weighted structural imaging was collected using a 3D MPRAGE (magnetization prepared rapid gradient-echo imaging) sequence with slightly different MR parameters among participants. The MR images acquired using Siemens scanner were scanned with the parameters as follows: repetition *time* (TR) = 2,300 ms, matrix = 240 × 256 × 176, slice thickness = 1.2 mm, and those parameters in General Electric scanner were as follows: TR = 7 ms, matrix = 256 × 256 × 166, slice thickness = 1.2 mm and those parameters in Philips scanner were as follows: TR = 6.8 ms, matrix = 256 × 256 × 170, slice thickness = 1.2 mm, respectively. More detailed information about the image acquisition procedures is available on the ADNI website^2^. Additionally, the MR data of Shanghai Mental Health Center were acquired using a Siemens Magnetom Verio 3.0 T scanner, and high-resolution T1-weighted structural images with 176 sagittal slices were collected using a MPRAGE sequence (TR = 2,530 ms, TE = 3.5 ms, flip angle = 9°, FOV = 256 mm × 256 mm, voxel size = 1.0 × 1.0 × 1.2 mm^3^).

### Imaging Preprocessing

Standardized preprocessing was necessary to improve discrimination between textural features and was performed using Statistical Parametric Mapping (SPM12) software^3^ implemented in MATLAB R2017a (The MathWorks, Natick, MA, USA). Firstly, each T1-weighted Digital Imaging and Communications in Medicine (DICOM) image was converted to Neuroimaging Informatics Technology Initiative (NIFTI) data. Secondly, correction for bias field inhomogeneities and intensity normalization of images were performed in the VBM12 toolbox. The corrected images were normalized to the Montreal Neurological Institute (MNI) standard T1 template (standard space 181 × 217 × 181 with a resolution of 1 mm × 1 mm × 1 mm) using DARTEL normalization. Then, the obtained images were spatially normalized to ensure that a given voxel corresponded to the same anatomical position in different subjects. Finally, we resliced those images to the standard MNI space with a resolution of 1 mm × 1 mm × 1 mm.

### Segmentation

Segmentation of the hippocampus was required to describe the texture characteristics of the region of interest (ROI). First, the bilateral hippocampus from the Anatomical Automatic Labeling (AAL) template provided by the MNI was chosen as the ROI mask. Then, the open-source software 3D-slicer^4^ was applied for medical image visualization and segmentation (Fedorov et al., 2012). Specifically, the viewer window of 3D-Slicer was used to select image “layers”, including “background” image and “label” image. Then, the standardized preprocessing image of each subject was loaded as the “background” image, and the left and right hippocampus mask was loaded as the“label” image, respectively. Next, two expert radiologists worked together to check the segmentation of the hippocampus for each subject and manually modified the unsatisfied image in the “Segment Editor” window of 3D slicer after reaching a consensus. In fact, a previous study has shown that the dice similarity coefficient (DSC) between the manual segmentation and atlas-based methods in brain structure segmentation are 0.79 (Ourselin et al., 2013). In our study, the combination of atlas-based segmentation and manual inspections could assure the segmentation quality and improve the time consumption.

### Feature Extraction

First, we loaded the standardized 3D T1-MPRAGE data for the EOAD, LOAD, YC, and OC subjects into 3D-slicer software, and then we imported the segmented left and right hippocampus. Massive features were selected using the “pyradiomics” package of the software^5^, including the histogram-based matrix (HISTO), GLCM, gray-level dependence matrix (GLDM), gray-level size zone matrix (GLSZM), GLRLM, and neighboring gray-tone difference matrix (NGTDM) in the “feature classes” window.

HISTO is a statistical description of discrete units, while the GLCM using second-order statistics reflects the spatial relationship of pixel gray-level values in the image (Dhruv et al., 2019). The GLDM is also based on the gray-level relationship to acquire the first-order statistics of local property values, and the GLRLM estimates the spatial relationships between groups of pixels with similar gray-level values (Araujo et al., 2018). The GLSZM can be used to compute different pixel distances, whereas the NGTDM measures the total differences in the gray level of a pixel (Thibault et al., 2014).

### Feature Selection

Before feature selection, preprocessing was required for accurate and valid selection. First, we checked the extracted data and replaced the abnormal values that deviated more than three standard deviations from the mean by the mean. Considering that deleting the abnormal values may cause loss of information and the lack of processing may affect the model construction, combined with the normal distribution of the data, we decided to replace outliers with the mean. Next, the subjects from ADNI data were randomly divided into training and test datasets at proportions of 0.7 and 0.3 for EOAD-YC groups, LOAD-OC groups, and EOAD-LOAD groups, respectively. Then, every extracted feature was standardized by the function of sklearn.preprocessing.scale based on Python programming to achieve Z-score normalization to remove the dimensional constraint.

We used Python programming to accomplish feature selection. First, t-test and Mann-Whitney U test were used to select the features with significant differences (*p* < 0.05). Next, correlation analysis was performed to further reduce the dimensionality. If the correlation coefficient of two feature columns exceeded 0.8, we removed one of them randomly. Finally, the least absolute shrinkage and selection operator (LASSO) regression analysis method with 10-fold cross validation was applied to determine the most valid features in the training data, and the corresponding lambda value was selected with minimum mean-squared error (MSE) values. The mechanism of LASSO, combining the penalty function and linear regression, makes some regression coefficients become zero and achieve dimension reduction (Tibshirani, 2013).

### Classification Analysis

Support vector machine (SVM) algorithms were used to construct radiomic models for EOAD and YCs, LOAD and OCs, and EOAD and LOAD. SVM is one of the most popular and mature machine learning algorithms based on the neuroimaging literature (Orru et al., 2012). The SVM model employs a radial basis function kernel using LIBSVM^6^ to implement nonlinear mapping from the input space to the feature space (Chang and Lin, 2011). Accordingly, the SVM models were used to construct the prediction models of the EOAD-YC groups, LOAD-OC groups, and EOAD-LOAD groups based on the selected prediction features in training sets, and then the test sets were used to calculate the predictive efficiency based on the predictive models, respectively (Nalepa and Kawulok, 2019). Then, all subjects from the data from Shanghai Mental Health Center were used as independent external validation sets to verify the reliability and robustness of the corresponding models. Additionally, receiver operating characteristic (ROC) curves and the corresponding areas under the curve (AUCs) were used to evaluate the diagnostic capabilities of the radiomic features.

### Statistical Analysis

Statistical analyses were performed using SPSS software 22.0 (IBM Corporation, Armonk, NY). The demographic information of the participants was collected as numbers or means ± SD for categorical and continuous variables. The comparisons between the EOAD and OC (EOAD-OC), LOAD and YC (LOAD-YC), and EOAD and LOAD (EOAD-LOAD) subjects were performed using χ^2^ test for categorical variables and Student’s t-test for continuous variables (two-tailed) to evaluate the differences between groups. A *p* < 0.05 was considered statistically significant.

## Results

### Demographic and Clinical Characteristics

The demographic and clinical characteristics of the four groups are presented in Table 1. No difference was found in age or sex between the EOAD patients and YCs (EOAD-YC) or between the LOAD patients and OCs (LOAD-OC) in the ADNI and SMHC data. The Mini-Mental State Examination (MMSE) scores were significantly different in the EOAD-YC and LOAD-OC groups (*p* < 0.001). No significant differences were found in the clinical dementia rating (CDR) scores and MMSE scores between the EOAD and LOAD patients (EOAD-LOAD).

**Table 1:** Demographic, clinical parameters for EOAD, LOAD, YC, and OC subjects.

	EOAD	YC	*p*	LOAD	OC	*p*	*P* (EOAD vs. LOAD)
**ADNI data**							
N	36	36		36	36		1
Age, y	59.80 ± 2.8	60.40 ± 2.4	0.31	72.45 ± 2.8	72.08 ± 1.4	0.48	<0.001
Gender, F(%)	18 (50%)	18 (50%)	1	19 (53%)	19 (53%)	1	0.81
CDR	0.8	-	-	0.8	-	-	1
MMSE	23.0 ± 1.6	29.0 ± 0.9	<0.001	22.5 ± 3.0	29.2 ± 0.4	<0.001	0.63
**SMHC data**							
N	15	15		15	15		1
Age, y	58.15 ± 5.4	59.85 ± 4.2	0.34	74.05 ± 5.8	73.51 ± 3.6	0.76	<0.001
Gender, F(%)	9 (60%)	9 (60%)	1	8 (53%)	8 (53%)	1	0.71
CDR	0.75	-	-	0.75	-	-	1
MMSE	22.1 ± 1.1	29.1 ± 0.7	<0.001	21.7 ± 1.8	28.3 ± 0.6	<0.001	0.47

*Values presented as mean ± standard deviation. EOAD, early-onset Alzheimer’s disease; LOAD, late-onset Alzheimer’s disease; YC, young control; OC, old control; CDR, Clinical Dementia Rating Scale; MMSE, Mini-Mental State Examination*.

### Feature Selection Results

**Figure 1:**
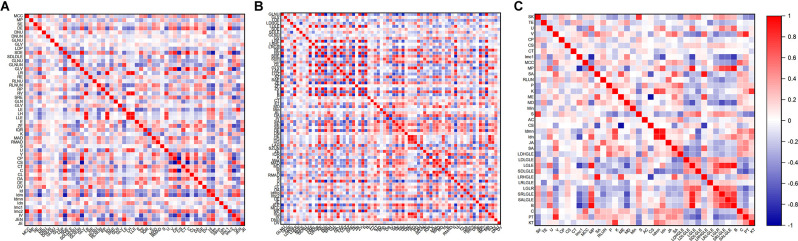
Correlation analysis graph of the EOAD-YC groups **(A)**, the LOAD-OC groups **(B)**, and the EOAD-LOAD groups **(C)**. EOAD, early-onset Alzheimer’s disease; LOAD, late-onset Alzheimer’s disease; YC, young control; OC, old control.

**Figure 2:**
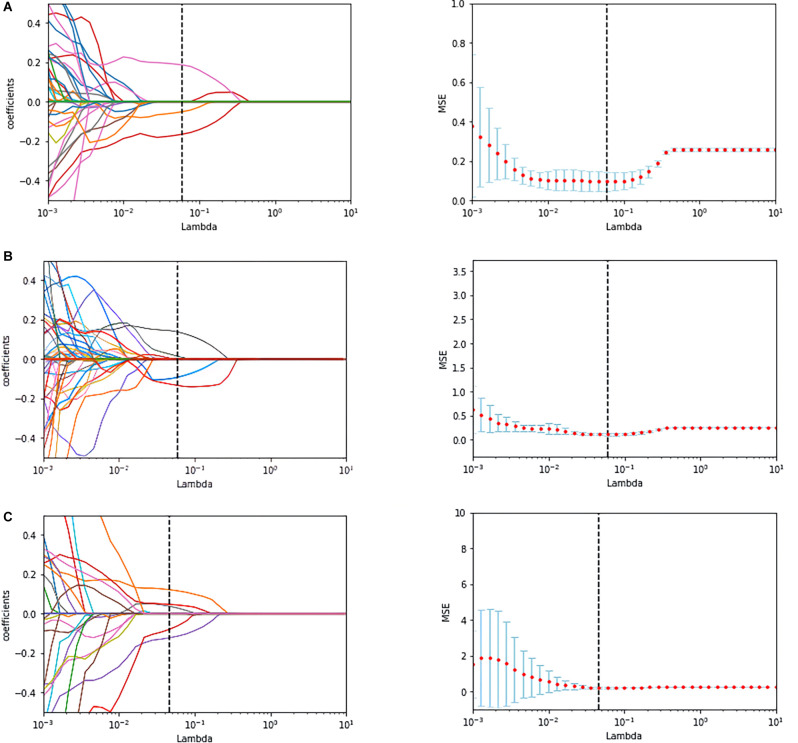
The coefficients-lambda graph and the MSE-lambda graph **(A)** in the EOAD-YC groups, the LOAD-OC groups **(B)**, and the EOAD-LOAD groups **(C)**. MSE, mean-squared error.

A total of 214 features were extracted from the bilateral hippocampus. After t-test and Mann-Whitney U test, 99, 102, and 37 features were preserved in the EOAD-YC, LOAD-OC, and EOAD-LOAD groups, respectively. After correlation analysis, 51, 73, and 24 (Figure 1) features remained. Finally, the LASSO regression model identified three, four, and four features for the EOAD-YC, LOAD-OC, and EOAD-LOAD groups (Table 2). Meanwhile, the values of the coefficients and the corresponding lambda values, and the MSE values and the corresponding lambda values for the EOAD-YC, LOAD-OC, and EOAD-LOAD groups are shown in Figure 2.

**Table 2:** The preserved radiomic features after the feature selection.

Type of features	EOAD-YC	LOAD-OC	EOAD-LOAD
Histogram	Kurtosis	Kurtosis Skewness	Kurtosis
GLCM	IMC1	IDMN	IDMN
GLDM		Dependence Entropy	Small Dependence Low Gray Level Emphasis
GLRLM			Long Run Low Gray Level Emphasis
NGTDM	Coarseness		

*GLCM, Gray-Level Co-Occurrence Matrix; GLDM, Gray Level Dependence Matrix; GLRLM, Gray-Level Run-Length Matrix; NGTDM, Neighbouring Gray Tone Difference Matrix; IMC1, Informational Measure of Correlation (IMC) 1; IDMN, Inverse difference moment normalized*.

### Classification Analysis Results

**Figure 3:**
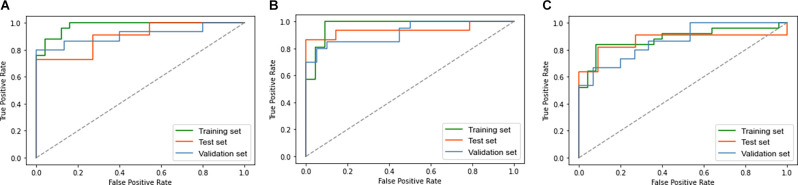
The ROC curve of the EOAD-YC groups in the training and test and validation sets **(A)**. The ROC curve of the LOAD-OC groups in the training and test and validation sets **(B)**. The ROC curve of the EOAD-LOAD groups in training and test and validation sets **(C)**. ROC, receiver operating characteristic.

The accuracy (ACC), sensitivity (SEN), specificity (SPE), and AUC were used to evaluate the classification performance. Figure 3 and Table 3 show the final classification performance on the test set and validation set. In the analysis between the EOAD patients and YCs, the ACC, SEN, SPE, and AUC were 0.77, 0.91, 0.64, and 0.90 in the test set and 0.87, 0.87, 0.87, and 0.91 in the validation set, respectively (Figure 3A). By contrast, in the LOAD patients and OCs, the ACC, SEN, SPE, and AUC were 0.86, 0.87, 0.86, and 0.94 in the test set and 0.78, 0.85, 0.70, and 0.92 in the validation set, respectively (Figure 3B). Finally, in the analysis between the EOAD and LOAD patients, the ACC, SEN, SPE, and AUC were 0.79, 0.67, 0.93, and 0.87 in the test set and 0.77, 0.60, 0.93, and 0.86 in the validation set, respectively (Figure 3C). Similar classification performance was found in the test and validation datasets, indicating that our models may have relatively good robustness.

**Table 3:** Classification performance on test and validation datasets.

		**Accuracy**	**Sensitivity**	**Specificity**	**AUC**
EOAD-YC	Training set	0.90	0.94	0.88	0.95
	Test set	0.77	0.91	0.64	0.90
	Validation set	0.87	0.87	0.87	0.91
LOAD-OC	Training set	0.91	0.96	0.82	0.97
	Test set	0.86	0.87	0.86	0.94
	Validation set	0.78	0.85	0.70	0.92
EOAD-LOAD	Training set	0.86	0.84	0.88	0.88
	Test set	0.79	0.67	0.93	0.87
	Validation set	0.77	0.60	0.93	0.86

AUC, areas under the curve.

## Discussion

The present study aimed to explore hippocampal radiomic features to distinguish between patients with EOAD and LOAD and healthy controls. Our findings show that the hippocampal radiomic-based classification model can discriminate patients with EOAD from YC subjects and distinguish LOAD patients from OC participants. Additionally, hippocampal texture was identified as a useful biomarker for LOAD and EOAD patients. Additionally, results from other datasets verified the generalizability and robustness of the models.

To our knowledge, this study is the first to construct a classification model of hippocampal radiomic features for EOAD patients and healthy subjects. This model reveals relatively good accuracy and sensitivity with a successful diagnostic value. Although EOAD patients account for 5–10% of reported AD cases (Lambert et al., 2014), this AD subtype is valuable to understand the underlying mechanism. Currently, studies on patients with EOAD have focused particularly on structural magnetic resonance imaging (sMRI; Yang et al., 2019). A quantitative analysis of the hippocampal volume in EOAD patients suggested that hippocampal atrophy has limited usefulness as a diagnostic biomarker for these patients (Falgas et al., 2019). Radiomic features, different from volumetric features, have captured considerable information and have shown great promise for personalized clinical applications (Avanzo et al., 2017). Our results show that the radiomic features of the hippocampus can be defined as a useful biomarker to identify EOAD patients and healthy controls, with great promise for personalized clinical application.

Our findings indicate that the hippocampal radiomic model presented excellent diagnostic value with good sensitivity and specificity to distinguish LOAD patients from OCs. Consistent with our results, radiomic analysis has been used to identify hippocampal features to distinguish LOAD patients from healthy control subjects. Chaddad et al. (2018) employed random forest (RF) models to identify hippocampal textural features to differentiate LOAD patients from normal controls (NCs) with an AUC of 0.84. Feng et al. (2018) demonstrated hippocampal radiomic features that distinguish LOAD patients from NCs with a classification accuracy of 0.87 *via* the SVM model. Luk et al. (2018) calculated a logistic regression model to classify LOAD patients and NCs, and the AUC was 0.93. Liu et al. (2018) achieved an AUC of 0.90 for classifying LOAD patients and NCs based on convolutional neural networks (CNNs). Furthermore, recent evidence suggests that hippocampal texture is significantly superior to hippocampal volumetry in the early detection of AD (Sorensen et al., 2016; Luk et al., 2018). Taken together, our findings support the significance of hippocampal textural features as promising neuroimaging biomarkers of AD.

Another important finding in this study worth noting is the relatively satisfying classification model of hippocampal radiomic features between EOAD and LOAD patients. This model has demonstrated relatively high specificity and accuracy with moderate diagnostic value. Notably, no radiomic analysis has investigated the radiomic features of brain regions to distinguish EOAD patients from LOAD patients directly. More recent attention has focused on neuroimaging analysis methods, including voxel-based morphometry (VBM), fMRI, diffusion tensor imaging (DTI), and multimodal MRI, to detect structural and functional changes in AD (Herdick et al., 2020). A recent structural MRI study revealed that compared with healthy controls, EOAD and LOAD patients exhibit a similar pattern of hippocampal atrophy (Eckerstrom et al., 2018). Therefore, it may be a challenge to distinguish between EOAD and LOAD relying on structural MRI. Radiomic analysis can extract and model many medical image features, and promises to increase precision in diagnosis and provide decision support for precision medicine (Lambin et al., 2017). Thus, radiomic studies of EOAD deserve higher priority. Our findings support the hypothesis that hippocampal radiomic features are valuable to distinguish the two types of AD.

Furthermore, in this study, three radiomic features were selected for the EOAD-YC groups—namely, kurtosis, coarseness, and informational measure of correlation 1 (IMC1). Kurtosis measures the degree of histogram sharpness, coarseness reflects the spatial rate of changes in gray-level intensities, and IMC1 captures the spatial relationships of pairs of pixels (Guiot et al., 2022). Concerning the LOAD-OC groups, kurtosis, skewness, inverse difference moment normalized (IDMN), and dependence entropy were filtered. Kurtosis and skewness are the parameters of the histogram, and skewness describes the degree of histogram asymmetry. IDMN describes texture homogeneity, whereas dependence entropy reflects the complexity in gray distribution (Salvatore et al., 2021). Additionally, four radiomic features—kurtosis, IDMN, small dependence low gray-level emphasis (SDLGLE), and long-run low gray-level emphasis (LRLGLE) were selected for the EOAD-LOAD groups. The first two features were consistent with the LOAD-OC groups. SDLGLE and LRLGLE are the parameters of GLDM and GLRLM, respectively. SDLGLE measures the joint distribution of small dependence with lower gray-level values, while LRLGLE evaluates the joint distribution of long run lengths with lower gray-level values (van Griethuysen et al., 2017). In summary, our results indicate differences and similarities in radiomic features among the EOAD-YC, LOAD-OC, and EOAD-LOAD groups.

This study has some limitations. First, owing to the relatively low prevalence rates for EOAD (Zhu et al., 2015), the limited sample size may affect the performance of the radiomic models. Second, the hippocampus is a heterogeneous structure encompassing different subregions, each of which may have distinct textural features (Blanken et al., 2017). Further studies regarding the radiomic features of hippocampal subregions are warranted. Finally, more longitudinal studies are needed combining texture with cerebrospinal fluid (CSF) and genomic and metabolic markers to achieve an accurate screening, diagnostic, and monitoring tool for clinical applications (Li et al., 2019).

## Conclusion

In conclusion, we found that hippocampal radiomic features can be used to distinguish patients with EOAD and LOAD from YCs and OCs. Furthermore, this study reports the moderately successful diagnostic classification of EOAD vs. LOAD based on hippocampal radiomic features. Generally, our findings support the possibility that hippocampal textural features may serve as potential neuroimaging biomarkers of AD, providing a useful tool for decision support in precision medicine.

## Data Availability Statement

The datasets presented in this study can be found in online repositories. The names of the repository/repositories and accession number(s) can be found below: http://adni.loni.usc.edu. The datasets from Shanghai Mental Health Center generated for this study are available on request to the corresponding author.

## Ethics Statement

The studies involving human participants were reviewed and approved by Shanghai Mental Health Center. The patients/participants provided their written informed consent to participate in this study. The use of the ADNI data was approved by the institutional review board at each site. Written informed consent was obtained from the individual(s) for the publication of any potentially identifiable images or data included in this article.

## Author Contributions

XL, YT, and YD conceived the study. YD, SZ, YF, QQ, LZ, and WW acquired and analyzed the data. YD and SZ drafted the manuscript. All authors contributed to the article and approved the submitted version.

## Conflict of Interest

The authors declare that the research was conducted in the absence of any commercial or financial relationships that could be construed as a potential conflict of interest.

## Publisher’s Note

All claims expressed in this article are solely those of the authors and do not necessarily represent those of their affiliated organizations, or those of the publisher, the editors and the reviewers. Any product that may be evaluated in this article, or claim that may be made by its manufacturer, is not guaranteed or endorsed by the publisher.

## Footnotes

^1^ http://adni.loni.usc.edu

^2^ http://adni.loni.usc.edu/methods/documents/

^3^ http://www.fil.ion.ucl.ac.uk/spm/software/spm12/

^4^ https://www.slicer.org/

^5^ http://www.jetbrains.com/pycharm/

^6^ http://www.csie.ntu.edu.tw/~cjlin/libsvm/index.html
